# Supplementary material for: Boundary complexity of cortical and subcortical areas predicts deep brain stimulation outcomes in Parkinson’s disease
Source: Nat Commun. 2025 Jul 1;16:5590. doi: 10.1038/s41467-025-60695-4 (PMC12219618; doi:10.1038/s41467-025-60695-4)
Supplement: Supplementary file 1 — Supplementary Information [file 41467_2025_60695_MOESM1_ESM.pdf]

Supplementary Information

Boundary complexity of cortical and subcortical areas predicts deep brain stimulation outcomes in Parkinson's disease

Devin Schoen[1,2], Skyler Deutsch[1], Juhi Mehta[1], Sarah Wang[3], John Kornak[4], Philip A. Starr[2,5], Doris D. Wang[2,5], Jill L. Ostrem[3], Ian O. Bledsoe[3], *Melanie A. Morrison[1,2]

[1]Department of Radiology and Biomedical Imaging, University of California San Francisco
[2]UCSF-UC Berkeley Joint PhD Program in Bioengineering
[3]Department of Neurology, University of California San Francisco
[4]Department of Epidemiology and Biostatistics, University of California San Francisco
[5]Department of Neurological Surgery, University of California San Francisco

Normality Testing

Methods: To ensure that the assumptions of normality were met for the residuals in our regression analyses, quantile-quantile (Q-Q) plots were generated to visually assess the distribution of residuals for all regions which were selected via LASSO regression in the main analysis. We also applied the Anderson-Darling test to quantitatively evaluate normality. A non-significant result from the Anderson-Darling test would indicate that the residuals do not significantly deviate from a normal distribution, thus supporting the assumption of normality for our regression models.

Results: All regions yielded non-significant results at the 1% significance level, with Anderson-Darling test statistics ranging from 0.295 to 1.150. These results indicate that we cannot reject the null hypothesis of normality for any region, suggesting that fractal dimension (FD) values in each region are approximately normally distributed.

Given the similarity in normality results across regions, a single Q-Q plot was generated to visually represent the pooled data, confirming a close alignment between the theoretical and sample quantiles (**Figure 1**). The consistency of these findings across all regions supports the assumption of normality, validating the use of parametric methods in the main analysis.

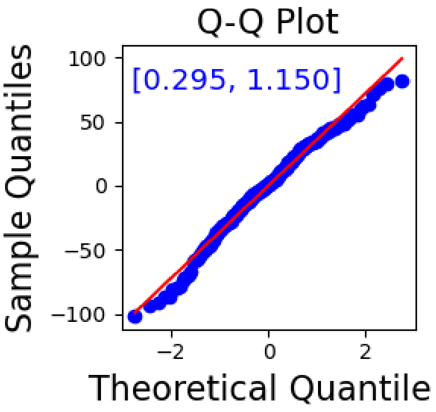

Figure 1: Quantile-quantile (Q-Q) plot of fractal dimension values pooled across brain regions, with Anderson-Darling statistics ranging from 0.295 to 1.150.

Fractal Dimension Reproducibility

Given that FD captures morphological complexity, it is essential to establish its stability across repeated imaging sessions. To assess the reproducibility of FD, we analyzed an openly available dataset from the Neuroimaging Tools and Research Collaboratory (NITRC), which includes T1-weighted MRI scans of healthy individuals acquired at multiple time points (Boekel et al., 2017). We examined short-term reproducibility by comparing FD values between two same-day scans acquired in separate imaging sessions. We also examined long-term reproducibility by comparing FD values from the first scan session to a follow-up scan acquired two weeks later.

<u>Methods:</u> Thirteen subjects with complete T1-w imaging data across all time points were included in the reproducibility analysis. FD was computed for 90 cortical and subcortical brain regions using the same automated processing pipeline described in the main manuscript. The first reproducibility test examined short-term stability by comparing FD values from two independent scan sessions on the same day, while the second test evaluated long-term reproducibility by comparing FD values from the initial session to a follow-up scan two weeks later.

Reproducibility was assessed using two complementary approaches. First, Pearson correlation coefficients (R^2) and linear regression were used to quantify the agreement between FD values across sessions. Second, a Bland-Altman analysis was performed to evaluate absolute agreement by calculating the mean difference (bias) between FD values across sessions and determining the 95% limits of agreement (LOA). The percentage of FD values falling within the LOA was computed to assess reproducibility.

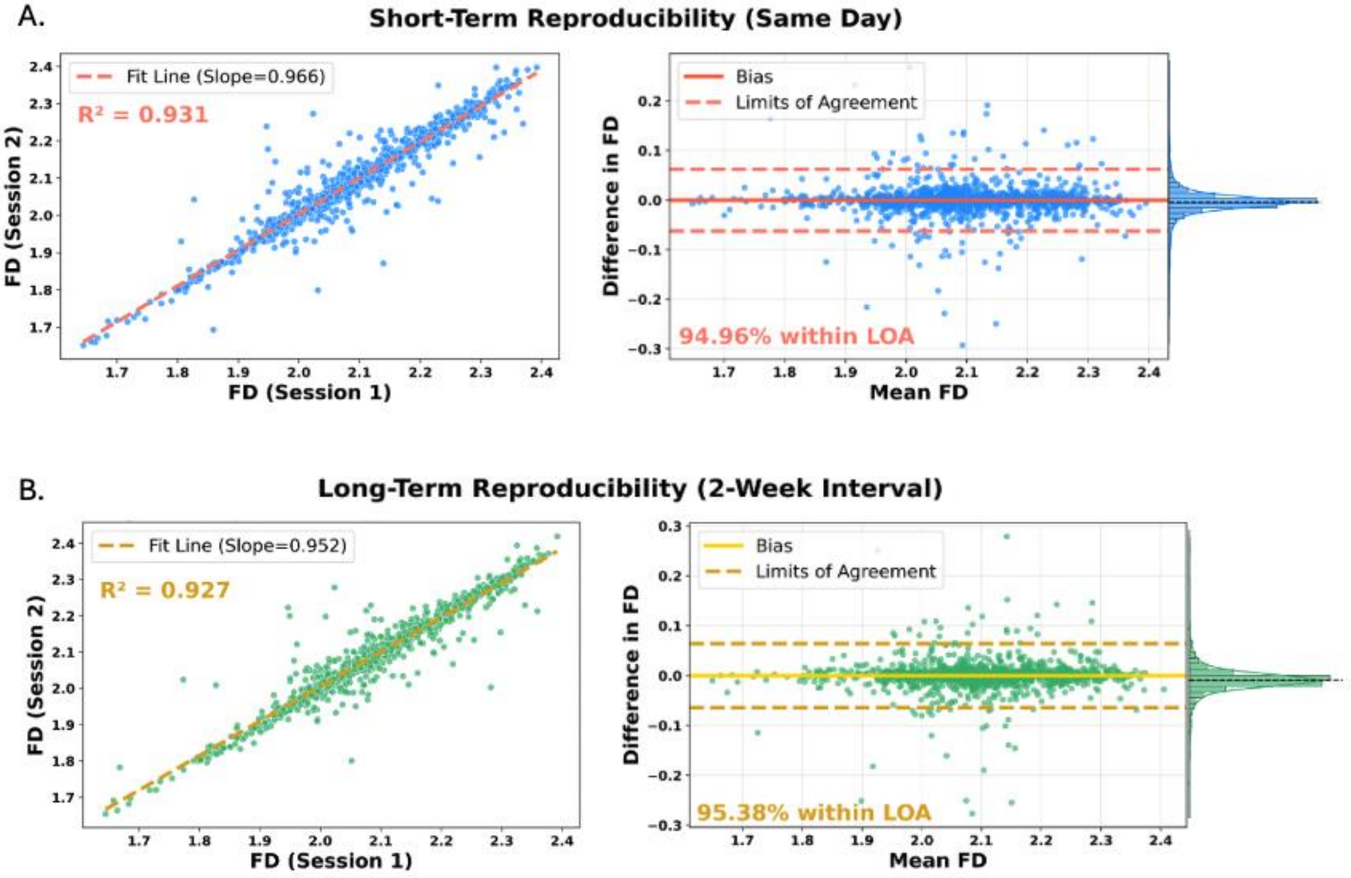

Figure 2: (A) Short-term reproducibility (same day): Scatter plot (left) of fractal dimension (FD) values between Session 1 and Session 2, with a linear fit and Pearson R^2. The Bland-Altman plot (right) shows the FD differences as a function of the mean FD, with the bias (solid line) and limits of agreement (dashed lines). (B) Long-term reproducibility (2-week interval): Same structure as (A), comparing Session 1 and the follow-up session after two weeks.
(n = 13 participants)

Results: Short-term reproducibility was high, as shown in **Figure 2A**. The Pearson correlation between Session 1 and Session 2 was strong (R^2=0.931), and the regression slope of 0.966 indicated minimal systematic bias between same-day FD measures. The Bland-Altman analysis further confirmed this stability, with a mean bias close to zero and 94.96% of FD values falling within the LOA, demonstrating that FD values remained highly consistent across short intervals.

Long-term reproducibility was similarly robust, as shown in Figure 2B. The correlation between Session 1 and the 2-week follow-up scan remained high (R^2=0.927), with a regression slope of 0.952, suggesting strong agreement over a longer period. Bland-Altman analysis showed a similar pattern, with 95.38% of FD values falling within the LOA, indicating that FD remains a stable metric over time.

Cross-Validation Feature Selection

In the main analysis, the dataset was split into training, validation, and testing sets at the very beginning of processing to ensure that feature selection was conducted independently of the test set. This separation was critical to avoid introducing biases into the final model evaluation, ensuring that selected features and model performance could be assessed on truly unseen data. This approach reflects a practical application where predictive models are applied to entirely new datasets. However, to further evaluate the robustness of selected features, here we conducted a complementary analysis using cross-validation. While cross-validation introduces overlap between feature selection and evaluation, making it unsuitable for the main analysis, it serves as an exploratory tool to assess feature importance across the full dataset and verify that the trends observed in the main analysis are consistent and stable. Features included in the selection process were the FD of 90 brain ROIs from the AAL atlas, preoperative medication responsiveness quantified as the percent improvement in MDS-UPDRS III motor scores from the OFF- to ON-medication state (%Δ MDS-UPDRS III), age, sex, DBS target (subthalamic nucleus or globus pallidus interna), and hemispheres treated (bilateral or unilateral).

<u>*Methods:*</u> Preprocessing involved standardizing all features using z-scores to ensure comparability. We implemented a 10-fold cross-validation framework, with the outer loop splitting the data into training and test sets. The stratified outer loop ensured that ΔLEDD remained balanced across folds. Within each outer fold, LASSO was applied with a fixed LASSO shrinkage factor (0.03 as selected in the main analysis) to perform feature selection on the training set, reducing dimensionality and isolating the most important predictors. Features with non-zero coefficients were considered selected for that fold.

To evaluate the consistency of feature selection, how often each feature was selected across the 10 outer folds was tracked. Cross-validation complements the main analysis by assessing trends in feature importance when the full dataset is used for both selection and evaluation. This approach allows us to validate the robustness of key features identified in the main analysis, ensuring that they are stable and biologically plausible. Although cross-validation does not replace the independent train-validation-test splitting method used in the main pipeline, it provides an additional exploratory lens to ensure the reliability of feature selection trends.

<u>*Results:*</u> This analysis identified the most frequently selected features across all folds (**Figure 3**). The cross-validation analysis and the main analysis revealed overlapping as well as distinct sets of features relevant to predicting ΔLEDD. Several features were consistently identified as important across both analyses. Notably, the left cingulate gyrus, posterior division, left parahippocampal gyrus, and right superior parietal gyrus appeared in both the cross-validation and original feature selection lists, emphasizing their robust contributions to the model. Additionally, clinical features such as DBS target, age, and preoperative motor response to PD

medications were repeatedly highlighted, underscoring the relevance of both anatomical and clinical predictors in explaining variability in the outcome.

However, there were also differences between the two approaches. The cross-validation analysis selected features such as the right calcarine cortex, right thalamus, and right angular gyrus, which were not identified in the original analysis. These regions may represent features that are sensitive to the inclusion of the entire dataset in feature selection, reflecting broader trends across all data splits. Conversely, the original analysis identified features like the left superior frontal gyrus and the left caudate nucleus, which were absent in the cross-validation results. These differences likely reflect the stricter separation of training, validation, and testing sets in the original analysis.

Despite these differences, the overlap between the two analyses supports the robustness of key anatomical and clinical predictors. The left posterior cingulate gyrus, identified in both analyses, is a region linked to motor control and cognitive processes, which are directly relevant to PD. The cross-validation analysis complements the main analysis by confirming trends in feature importance while highlighting additional features that might warrant further investigation in future studies.

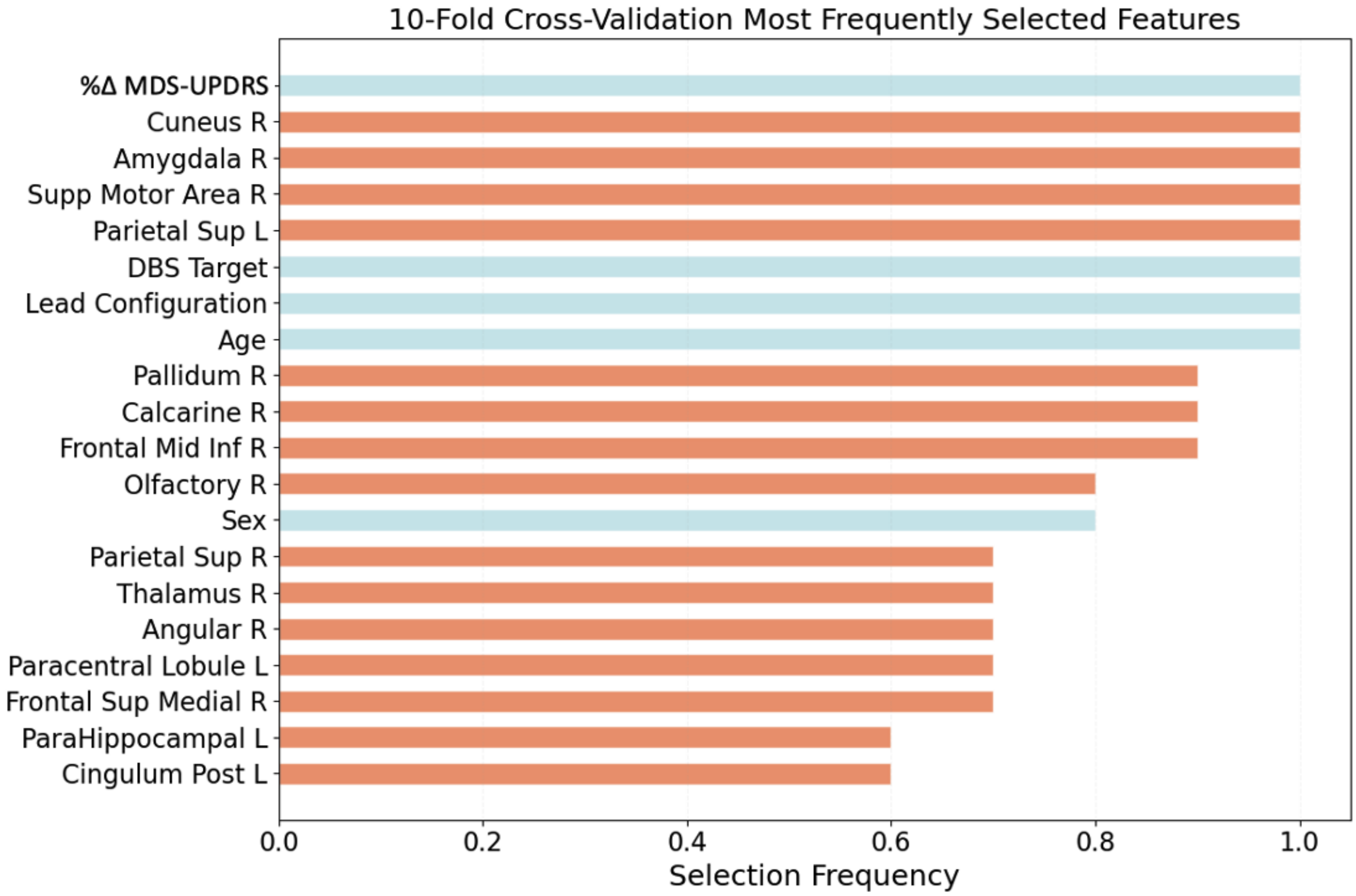

Figure 3: Features most frequently selected in the cross-validation analysis using LASSO regression, aggregated across 10 folds. Fractal dimension features are shown in orange, while clinical variables are shown in blue.
