# Supplementary material for: Boundary complexity of cortical and subcortical areas predicts deep brain stimulation outcomes in Parkinson’s disease
Source: Nat Commun. 2025 Jul 1;16:5590. doi: 10.1038/s41467-025-60695-4 (PMC12219618; doi:10.1038/s41467-025-60695-4)
Supplement: Supplementary file 2 — Reporting Summary [file 41467_2025_60695_MOESM2_ESM.pdf]

nature portfolio

Corresponding author(s): Melanie Morrison

Last updated by author(s): May 29, 2025

Reporting Summary

Nature Portfolio wishes to improve the reproducibility of the work that we publish. This form provides structure for consistency and transparency in reporting. For further information on Nature Portfolio policies, see our Editorial Policies and the Editorial Policy Checklist.

Statistics

For all statistical analyses, confirm that the following items are present in the figure legend, table legend, main text, or Methods section.

n/a	Confirmed	
☐	☒	The exact sample size (n) for each experimental group/condition, given as a discrete number and unit of measurement
☒	☐	A statement on whether measurements were taken from distinct samples or whether the same sample was measured repeatedly
☐	☒	The statistical test(s) used AND whether they are one- or two-sided<br>*Only common tests should be described solely by name; describe more complex techniques in the Methods section.*
☐	☒	A description of all covariates tested
☐	☒	A description of any assumptions or corrections, such as tests of normality and adjustment for multiple comparisons
☐	☒	A full description of the statistical parameters including central tendency (e.g. means) or other basic estimates (e.g. regression coefficient) AND variation (e.g. standard deviation) or associated estimates of uncertainty (e.g. confidence intervals)
☐	☒	For null hypothesis testing, the test statistic (e.g. F, t, r) with confidence intervals, effect sizes, degrees of freedom and P value noted<br>*Give P values as exact values whenever suitable.*
☒	☐	For Bayesian analysis, information on the choice of priors and Markov chain Monte Carlo settings
☒	☐	For hierarchical and complex designs, identification of the appropriate level for tests and full reporting of outcomes
☐	☒	Estimates of effect sizes (e.g. Cohen's d, Pearson's r), indicating how they were calculated

Our web collection on statistics for biologists contains articles on many of the points above.

Software and code

Policy information about availability of computer code

Data collection	T1-weighted MRI data were retrospectively collected from multiple MRI scanners across different institutions over a decade. Data acquisition involved various commercial MRI systems (e.g., Siemens, Philips, GE Healthcare) and their proprietary acquisition software, though the specific versions are not available due to the retrospective nature of the study.
Data analysis	Data analysis involved the use of a combination of open-source software, in-house code, and established Python packages:<br>- Image Processing: FSL (version 6.0.6.4) and MRTrix3 (version 3.0.4) were used for image preprocessing, including registration and diffusion processing. Skull stripping was performed using HD-BET, an open-source tool for brain extraction (version 1.0).<br>- Fractal Dimension Calculation: In-house Python code was used to compute fractal dimensions from T1-weighted images. Key libraries included nibabel, matplotlib, numpy, plotly, and Python's math module for image handling, visualization, and mathematical computations.<br>- Hypergraph Construction and Classification: Pytorch (version 1.13.1) and the open-source Pytorch package DHG (Deep Hypergraph) were used for building hypergraph structures and performing classification tasks.<br>- Statistical Analysis and Visualization: Python packages such as scipy, pandas, seaborn, and statsmodels were used for statistical analyses, and matplotlib and plotly were used for creating visualizations.

For manuscripts utilizing custom algorithms or software that are central to the research but not yet described in published literature, software must be made available to editors and reviewers. We strongly encourage code deposition in a community repository (e.g. GitHub). See the Nature Portfolio guidelines for submitting code & software for further information.

Data

Policy information about availability of data

All manuscripts must include a data availability statement. This statement should provide the following information, where applicable:

- Accession codes, unique identifiers, or web links for publicly available datasets
- A description of any restrictions on data availability
- For clinical datasets or third party data, please ensure that the statement adheres to our policy

Example data and processing code used in this study are available on our lab's GitHub repository at https://github.com/Radiology-Morrison-lab-UCSF/T1w_FractalDimension. The raw T1-weighted MRI data and associated clinical information used in this study are currently not publicly available due to ongoing efforts to ensure participant privacy, proper deidentification (e.g., removal of identifiable facial features), and consent for data sharing. Once these processes are complete, the data will be uploaded to OpenNeuro and made publicly available, with the corresponding accession code added to the GitHub repository.

Research involving human participants, their data, or biological material

Policy information about studies with human participants or human data. See also policy information about sex, gender (identity/presentation), and sexual orientation and race, ethnicity and racism.

Reporting on sex and gender	This study included 155 males and 76 females, reflecting the known sex disparity in Parkinson's disease prevalence, which disproportionately affects males. Sex data were obtained from clinical records. In cases where gender differed from sex, only sex was used for the purposes of this study. Sex was included as a covariate in our analyses to account for its potential influence on the results. In the final LASSO regression feature selection, sex was not identified as one of the most informative predictors of outcomes. However, sex remains an important factor in Parkinson's disease research due to its association with differential disease progression, symptomatology, and treatment response. Disaggregated sex-based data are not included in this study's source data.
Reporting on race, ethnicity, or other socially relevant groupings	Race and ethnicity data were not collected for this study, which is a potential limitation. While race may not be broadly relevant to Parkinson's disease prevalence or progression, it could have implications for deep brain stimulation (DBS) outcomes, potentially due to differences in access to care, treatment responses, or other socio-demographic factors. However, since this information was not available in our dataset, race and ethnicity were not included as covariates or considered in our analyses. Future studies should prioritize collecting this information to better understand its potential influence on DBS outcomes and ensure equitable healthcare research.
Population characteristics	The primary analysis included 231 patients with Parkinson's disease (PD) who underwent deep brain stimulation (DBS) at UCSF. Covariate-relevant characteristics included age (mean ± SD: 65 ± 9 years), DBS hemisphere laterality (unilateral: 44; bilateral: 187), and DBS target (Globus Pallidus Internus [GPi]: 135; Subthalamic Nucleus [STN]: 96). These covariates were included in the statistical analysis to account for their potential influence on the study outcomes.<br>A sub-analysis to evaluate whether fractal dimension can distinguish between PD patients and healthy controls used a subset of the primary cohort along with publicly available datasets from five additional repositories. This included data from three healthy control sources and two PD sources, with the groups being age-matched to minimize potential confounding effects.
Recruitment	Participants were recruited retrospectively from the UCSF Deep Brain Stimulation (DBS) Center. Patients were contacted by phone by a Clinical Research Coordinator and asked for consent to use their imaging and clinical data for research purposes. Inclusion criteria required that participants have T1-weighted images of sufficient quality and pre- and post-operative levodopa equivalent daily dose (LEDD) measurements available to calculate the delta LEDD outcome. Only patients who were of sound enough mind to provide informed consent were included, and no new data were collected, meaning participants did not need to attend additional visits.<br>Potential selection bias may exist due to variability in patient response rates, as those with poorer experiences or outcomes with DBS might have been less likely to respond or agree to participate. This could potentially impact the generalizability of the findings by skewing the cohort toward participants with more favorable outcomes or perceptions of DBS.
Ethics oversight	This study was approved by the Institutional Review Board (IRB) at the University of California, San Francisco (UCSF). All research procedures adhered to the ethical guidelines set forth by the IRB, and informed consent was obtained from all participants prior to the use of their data for research purposes.

Note that full information on the approval of the study protocol must also be provided in the manuscript.

Field-specific reporting

Please select the one below that is the best fit for your research. If you are not sure, read the appropriate sections before making your selection.

☒ Life sciences ☐ Behavioural & social sciences ☐ Ecological, evolutionary & environmental sciences

For a reference copy of the document with all sections, see nature.com/documents/nr-reporting-summary-flat.pdf

Life sciences study design

All studies must disclose on these points even when the disclosure is negative.

Sample size	To ensure that the study was adequately powered, a power analysis was conducted to estimate the minimum number of subjects required. The goal was to achieve 90% power at a 0.05 significance level for detecting a meaningful effect of imaging feature predictors on LEDD outcomes. Based on preliminary data, an effect size of approximately 0.3 was calculated from differences between groups of patients whose LEDD either increased or decreased after treatment. This analysis determined that a minimum of 123 subjects was required.<br>Our cohort of 231 patients, which includes imaging and outcome metrics, far exceeds this requirement, ensuring robustness and reliability in the analyses. This sample size is one of the largest for studies of this kind, providing a strong foundation for detecting statistically and clinically meaningful effects.
Data exclusions	no data was excluded
Replication	Measures were taken to verify the reproducibility of the experimental findings. The predictive models were tested with different random generator seeds and alternative data splits, and model performance remained consistent across these variations. To further assess reproducibility, a 10-fold cross-validation was conducted for feature selection, and similar features were selected as in the main analysis, with some differences. Features selected most frequently across the 10 folds were prioritized for interpretation.<br>In the main analysis, data were split into training, validation, and testing sets. Feature selection was performed exclusively on the training set to ensure that model optimization and testing remained unbiased. The observed differences in feature selection between the 10-fold cross-validation and the main analysis are consistent with this approach and reflect expected variability, though there was substantial overlap in selected features.
Randomization	Participants were allocated into experimental groups using randomization methods implemented in Python. Due to the uneven distribution of groups based on whether the levodopa equivalent daily dose (LEDD) decreased or did not decrease (with a higher proportion experiencing a reduction in medication burden), the data splitting was stratified by LEDD change. This ensured that both groups were proportionally represented across the training, validation, and testing sets, helping to control for potential imbalances.
Blinding	Blinding was not relevant to this study as it involved retrospective data analysis of pre-existing imaging and clinical data. Group allocation (e.g., based on changes in levodopa equivalent daily dose [LEDD]) was determined after data collection and was intrinsic to the analysis. As such, blinding of investigators during data collection or analysis was not applicable.

Reporting for specific materials, systems and methods

We require information from authors about some types of materials, experimental systems and methods used in many studies. Here, indicate whether each material, system or method listed is relevant to your study. If you are not sure if a list item applies to your research, read the appropriate section before selecting a response.

Materials & experimental systems

n/a	Involved in the study	
x		Antibodies
x		Eukaryotic cell lines
x		Palaeontology and archaeology
x		Animals and other organisms
x		Clinical data
x		Dual use research of concern
x		Plants

Methods

n/a	Involved in the study	
x		ChIP-seq
x		Flow cytometry
	x	MRI-based neuroimaging

Plants

Seed stocks	*Report on the source of all seed stocks or other plant material used. If applicable, state the seed stock centre and catalogue number. If plant specimens were collected from the field, describe the collection location, date and sampling procedures.*
Novel plant genotypes	*Describe the methods by which all novel plant genotypes were produced. This includes those generated by transgenic approaches, gene editing, chemical/radiation-based mutagenesis and hybridization. For transgenic lines, describe the transformation method, the number of independent lines analyzed and the generation upon which experiments were performed. For gene-edited lines, describe the editor used, the endogenous sequence targeted for editing, the targeting guide RNA sequence (if applicable) and how the editor was applied.*
Authentication	*Describe any authentication procedures for each seed stock used or novel genotype generated. Describe any experiments used to assess the effect of a mutation and, where applicable, how potential secondary effects (e.g. second site T-DNA insertions, mosiacism, off-target gene editing) were examined.*

Magnetic resonance imaging

Experimental design

Design type	this study doesn't include function MRI
Design specifications	this study doesn't include function MRI
Behavioral performance measures	this study doesn't include function MRI

Acquisition

Imaging type(s)	structural T1-weighted MR
Field strength	3.0 T
Sequence & imaging parameters	The T1-w imaging protocols had variable acquisition parameters reflecting real-world clinical settings, including repetition times=680-1000ms, echo times=3-4ms, field-of-view=256x256mm, and voxel resolutions of 0.5x0.5x1mm or 1mm isotropic.
Area of acquisition	whole brain scan

Diffusion MRI ☐ Used ☒ Not used

Preprocessing

Preprocessing software	FSL (version 6.0.6.4) and MRTrix3 (version 3.0.4) were used for image preprocessing, including registration and diffusion processing. Skull stripping was performed using HD-BET, an open-source tool for brain extraction (version 1.0).
Normalization	T1-weighted MRI images were normalized to MNI space using both linear and non-linear registration methods. First, an affine transformation was applied using FLIRT to align the skull-stripped images with the MNI template. Subsequently, FNIRT was used for non-linear registration to refine alignment and account for anatomical variability across subjects. ROIs were transformed back to subject space using the inverse of the warp coefficients calculated during non-linear registration.
Normalization template	MNI152NLin2009bAsym
Noise and artifact removal	To address structural noise, skull stripping was performed on T1-weighted images using the HD-BET tool to remove non-brain tissues such as the skull and scalp. Spatial misalignment artifacts were minimized through affine and non-linear registration to the MNI template using FLIRT and FNIRT, respectively. Intermediate outputs were visually inspected during processing to ensure quality and identify potential artifacts.<br>Motion parameters, physiological signals, or other sources of noise were not applicable to this study, as it focused on retrospective analysis of structural T1-weighted MRI data, which is inherently less affected by these artifacts compared to functional or diffusion imaging.
Volume censoring	Volume censoring is not applicable to this study, as it involved structural T1-weighted MRI data rather than time-series imaging modalities such as fMRI. Quality assurance steps, including visual inspection and skull stripping, were performed to ensure data quality and minimize artifacts.

Statistical modeling & inference

Model type and settings	This study employed a combination of predictive modeling and variance analysis. The main predictive models were based on LASSO regression and Hypergraph Neural Networks (HGNNs) to evaluate the relationship between fractal dimension (FD) and Parkinson's disease (PD) deep brain stimulation (DBS) outcomes.<br>LASSO regression was used for feature selection from a high-dimensional dataset, prioritizing predictors most strongly associated with LEDD change. LASSO penalization (alpha = 0.03) was optimized via cross-validation to minimize overfitting.<br>Ridge regression was then applied to the selected features to address multicollinearity and estimate normalized feature coefficients.<br>Hypergraph-based classification learning models were constructed to integrate clinical and imaging features. The HGNN framework captured high-order relationships by defining hyperedges connecting subjects with similar features. These models were evaluated using an area under the receiver operating characteristic curve (AUC).
Effect(s) tested	The primary effect tested was the relationship between selected features (clinical and fractal dimension metrics) and DBS outcomes, specifically changes in levodopa equivalent daily dose (ΔLEDD). Analyses tested whether the inclusion of FD features improved model fit and predictive accuracy compared to clinical features alone.<br>Variance analysis using Ordinary Least Squares (OLS) regression compared models with clinical features alone versus combined clinical and FD features. An F-test was used to assess whether the additional variance explained by FD was statistically significant.<br>HGNN models were used to classify patients based on LEDD outcomes (increase vs. decrease/no change). Classification performance was compared between models using clinical features only and those integrating FD metrics.<br>A separate analysis evaluated target-specific effects (STN vs. GPi) by including interaction terms between FD and DBS target in OLS regression.

Specify type of analysis: ☐ Whole brain ☒ ROI-based ☐ Both

Anatomical location(s): Anatomical locations were determined using the Automated Anatomical Labeling (AAL) atlas. This probabilistic atlas was used to segment the brain into 90 predefined regions of interest (ROIs). The atlas was registered to each subject's native T1-weighted MRI space using affine and non-linear transformations via FSL (FLIRT and FNIRT). After transformation, the inverse warp was applied to map the AAL ROIs to the subject's brain. Regional fractal dimension (FD) was calculated for each ROI in the native space to ensure alignment with individual anatomical variability.

Statistic type for inference: This study employed ROI-based analysis rather than voxel-wise or cluster-wise methods.

(See Eklund et al. 2016)

Correction: Correction for multiple comparisons was applied where appropriate, using the Benjamini-Hochberg false discovery rate (FDR) method.

Models & analysis

n/a	Involved in the study
☒	☐ Functional and/or effective connectivity
☒	☐ Graph analysis
☐	☒ Multivariate modeling or predictive analysis

Multivariate modeling and predictive analysis: -Independent Variables: Clinical features (age, sex, DBS target, lead laterality, and levodopa responsiveness) and fractal dimension (FD) values from 90 brain regions defined by the AAL atlas.
- Feature Extraction and Dimension Reduction: FD values were calculated using the box-counting method. LASSO regression was used for feature selection, and cross-validation ensured stability across data splits.
- Model: Ridge regression assessed feature importance for ΔLEDD, and Hypergraph Neural Networks (HGNNs) integrated clinical and FD features for classification.
- Training and Evaluation Metrics: Data were split into training, validation, and testing sets. Ridge regression performance was evaluated using R-squared values and F-tests. HGNN classification performance was assessed via AUC, sensitivity, and specificity, with FD features improving AUC from 0.64 to 0.76.
